# Organotypic culture in three dimensions prevents radiation-induced transformation in human lung epithelial cells

**DOI:** 10.1038/srep31669

**Published:** 2016-08-19

**Authors:** Mariam El-Ashmawy, Melissa Coquelin, Krishna Luitel, Kimberly Batten, Jerry W. Shay

**Affiliations:** 1 Department of Cell Biology, University of Texas Southwestern Medical Center, Dallas, Texas, 75235, United States of America

## Abstract

The effects of radiation in two-dimensional (2D) cell culture conditions may not recapitulate tissue responses as modeled in three-dimensional (3D) organotypic culture. In this study, we determined if the frequency of radiation-induced transformation and cancer progression differed in 3D compared to 2D culture. Telomerase immortalized human bronchial epithelial cells (HBECs) with sh*TP53* and mutant *KRas* expression were exposed to various types of radiation (gamma, ^+^H, ^56^Fe) in either 2D or 3D culture. After irradiation, 3D structures were dissociated and passaged as a monolayer followed by measurement of transformation, cell growth and expression analysis. Cells irradiated in 3D produced significantly fewer and smaller colonies in soft agar than their 2D-irradiated counterparts (gamma *P* = 0.0004; ^+^H *P* = 0.049; ^56^Fe *P* < 0.0001). The cell culture conditions did not affect cell killing, the ability of cells to survive in a colony formation assay, and proliferation rates after radiation—implying there was no selection against cells in or dissociated from 3D conditions. However, DNA damage repair and apoptosis markers were increased in 2D cells compared to 3D cells after radiation. Ideally, expanding the utility of 3D culture will allow for a better understanding of the biological consequences of radiation exposure.

Although radiation therapy is a common treatment for cancer patients, ionizing radiation (IR) also damages DNA and cellular components in healthy cells, leading to carcinogenesis and cell death^1^,^2^. As a transforming agent, radiation exposure has been linked to secondary cancers later in life^3^,^4^, and heavy particle-induced carcinogenesis is still a major hurdle for long-term space flights^5^.

Our ability to accurately assess human cancer risks, especially in the lung, is limited by a lack of good *in vitro* models. Most prediction models of radiation-induced cancers are based on studies performed with cells outside their normal biological context. Extracellular matrix, mesenchymal cells such as fibroblasts, endothelial and smooth muscle cells are lost when cells are grown under artificial conditions (2D). However, these microenvironmental factors play a critical role in cell growth, polarity, structural organization, signaling, and cell fate in whole tissues under normal physiologic conditions^6^,^7^,^8^.

The use of three-dimensional (3D) cell culture systems has greatly broadened the scope of culture methods and contributed to narrowing the gap between *in vitro* and *in vivo* research^9^. Characterization of differences in radiation effects between 2D monolayer and 3D cell cultures suggests cells cultured in 3D extracellular matrix are more radio- and chemoresistant than cells grown under conventional 2D conditions^10^,^11^. This has been partly explained by increased levels of heterochromatin in 3D cultures, thus reducing the number of DNA breaks and lethal chromosomal aberrations in 3D-grown tumor cells^12^. Integrin-mediated cell–matrix interactions, cell shape, nuclear organization and chromatin structure have all been implicated in the differential effect in cull culture^10^. However, not all radiation experiments using 3D cell cultures have shown differences in cell death, damage, or chromosomal aberrations, indicating that the tissue type and exact 3D culture method may be highly influential^13^.

To better simulate physiological architecture and understand lung responses, 3D culture models have been established using human bronchial epithelial cells (HBECs)^14^,^15^,^16^,^17^. When cultured in various 3D conditions, HBECs are able to differentiate into multiple airway cells types^18^,^19^,^20^, and cultured on top of basement membrane-like Matrigel overlaying lung fibroblasts, HBECs form web-like aggregates that branch and bud resembling the lung during development^21^. Since HBECs grown in 3D culture appear to form higher order, differentiated cellular structures similar to native lung physiology compared to the same cells grown in 2D culture, 3D cells may be a more accurate model for assessing the effects of radiation on cancer progression and transformation in the lung. We determined if 3D culture affects radiation-induced transformation or subsequent repair pathways when compared to radiation in standard 2D culture.

## Results

### 3D-irradiated cells are less invasive compared to 2D-irradiated cells

**Figure 1:**
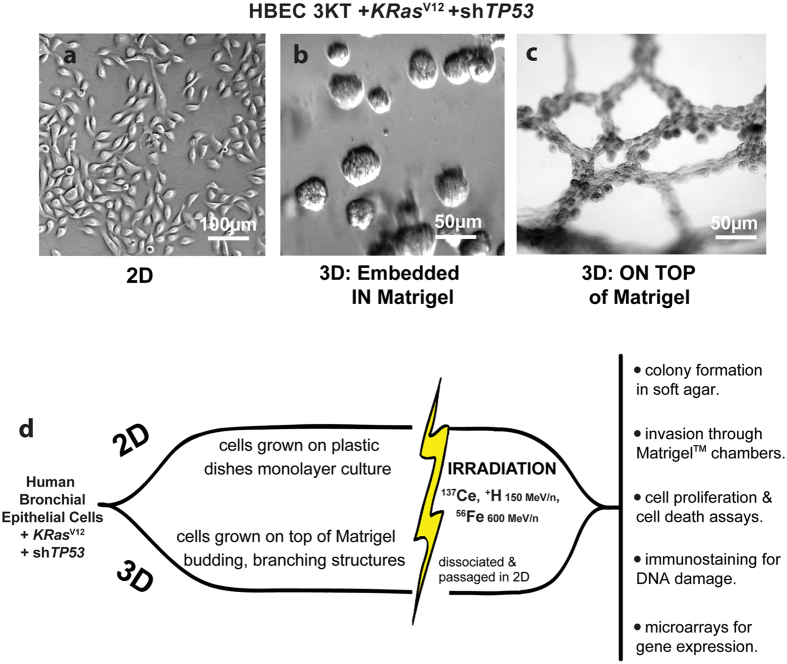
Timeline and design of experimental conditions. HBEC cells grown in (**a**) 2D in KSFM, (**b**) 3D embedded in a Matrigel suspension, and (**c**) 3D grown on top of Matrigel. (**d**) Schematic of experimental design. Once 3D cells formed branching structures (~5 days), both 2D and 3D culture conditions were exposed to different types of radiation. Different experimental endpoints are listed, which either occur immediately (24 h), or soon (within three population doublings) after IR exposure.

**Figure 2:**
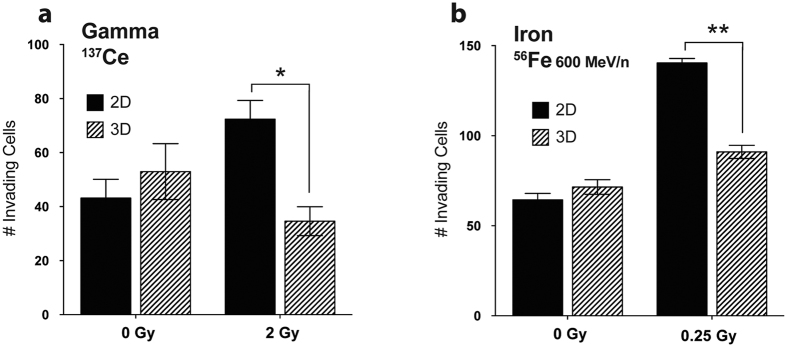
Invasion through Matrigel chambers soon after IR exposure. The average number of invading cells per field after irradiation with (**a**) 2Gy γ or (**b**) 0.25Gy iron (600 MeV/n). Only 2D-irradiated cells have increased numbers of invading cells. *P = 0.0015, **P < 0.0001; mean ± SEM.

To assess the ability of cells to experimentally migrate and invade through basement membrane, 2D and 3D cell cultures [Fig. 1a,c] exposed to γ or iron radiation were seeded in Matrigel invasion chambers [Fig. 1d]. 3D cells exposed to γ or iron had significantly fewer invading cells than 2D-irradiated cells (^*^P = 0.0015, **P < 0.0001) [Fig. 2]. Thus, cells irradiated under 3D conditions have reduced invasive properties compared to cells irradiated in 2D culture.

### Cells irradiated in 3D form fewer and smaller colonies in soft agar compared to cells irradiated in 2D

**Figure 3:**
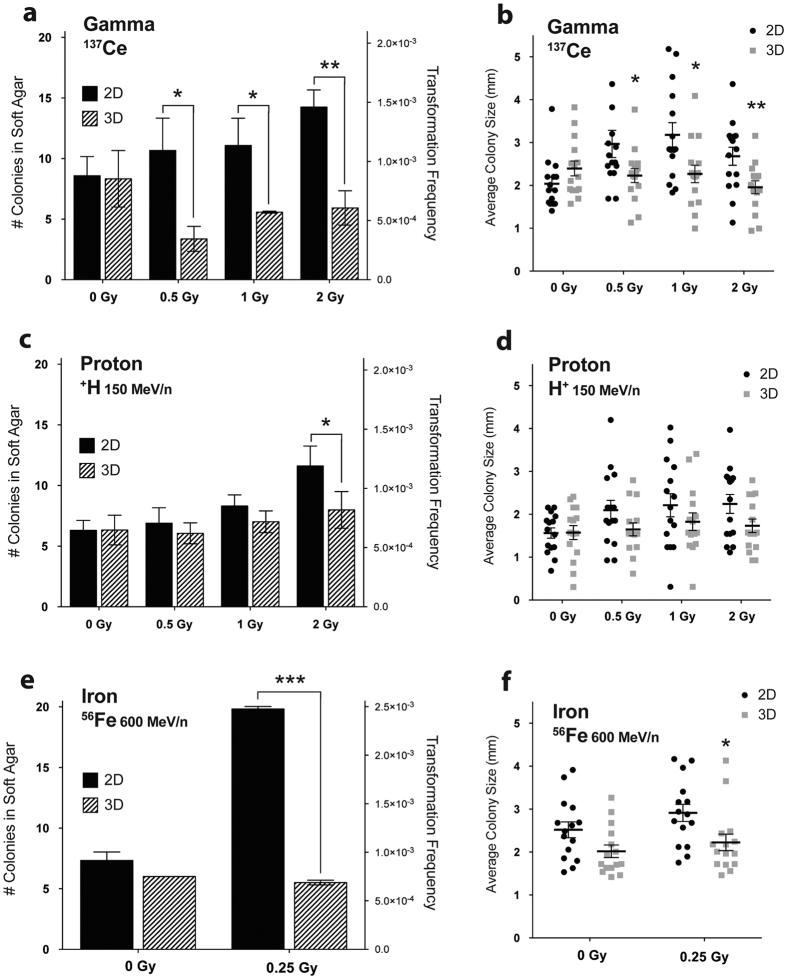
Number and size of soft agar colonies soon after exposure to IR. Anchorage independent colony growth soon after irradiation with (**a,b**) γ (**C,D**) proton, and (**e,f**) iron shows 3D-irradiated cells form fewer and smaller colonies than 2D-irradiated cells. *P < 0.05, **P < 0.01, ***P < 0.0001; mean ± SEM.

**Figure 4:**
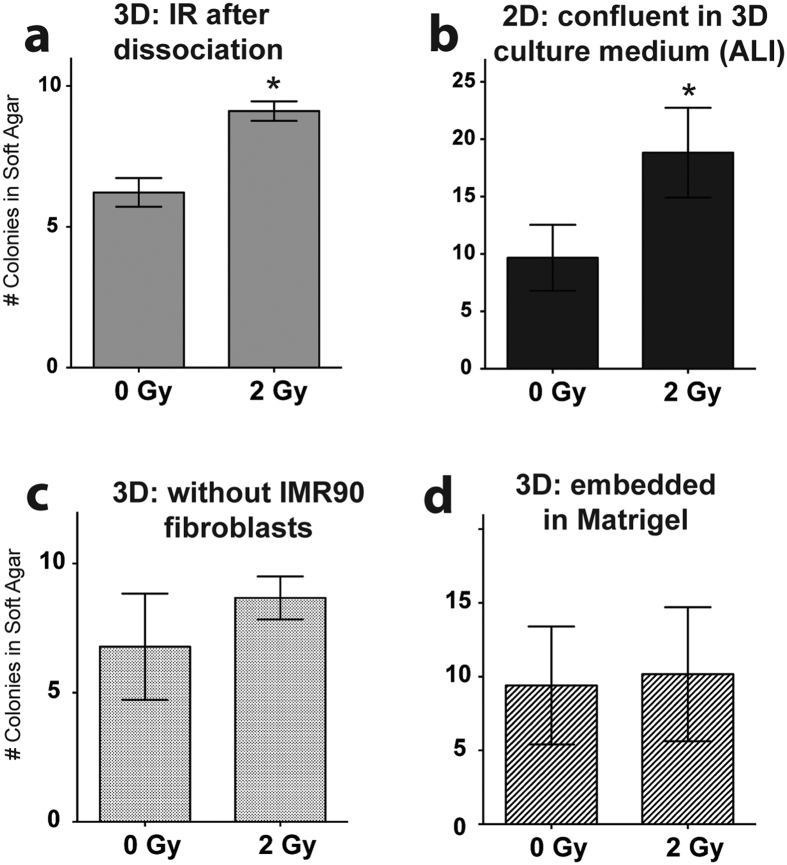
Alternative and intermediate culture conditions recapitulate soft agar phenotype. Various permutations of 2D and 3D cultures were irradiated with 2Gy γ. (**a**) HBECs were grown on top of 3D Matrigel culture (exactly like Fig. 1c), and were only irradiated after being dissociated from 3D culture and forming a 2D monolayer again. *P = 0.05; mean ± SEM. (**b**) HBECs were grown to confluency in ALI medium, then irradiated. Cells retain increased growth in soft agar after IR. *P = 0.04; mean ± SEM. (**c**) HBECs were grown on top of 3D Matrigel without using a fibroblast feeder layer. (**d**) HBECs were embedded in a 90:10 Matrigel:ALI suspension on top of a feeder layer, which also prevents the increase in radiation-induced anchorage independent growth. Mean ± SEM.

3D structures were dissociated after IR and cultured as a monolayer alongside 2D before being seeded into soft agar assays [Fig. 1d]. Basal soft agar transformation frequency for HBEC 3KT+*KRas*^v12^+sh*TP53* is approximately nine cells per 10,000 regardless of their initial culture conditions [see Supplemental Figure 1]. With increasing doses of γ exposure, there is dose-dependent increase in the number of anchorage-independent colonies in cells exposed in 2D (0Gy to 2Gy; *P* < 0.05) [Fig. 3a]. However, 3D-irradiated cells formed significantly fewer colonies compared to their 2D-irradiated counterparts (^*^P = 0.018, *P = 0.023, **P = 0.0004) [Fig. 4a]. Also, the size of these 3D colonies was smaller on average compared to 2D colonies (*P = 0.048, *P = 0.014, **P = 0.0095) [Fig. 3b]. This pattern held true with protons at 2Gy at 150 MeV/n (*P = 0.049) [Fig. 3c], although colony sizes were not significantly different [Fig. 3d]. The highest transformation frequency was in 2D with iron (0.25 Gy at 600 Mev/n), with cells forming significantly more colonies compared to unirradiated control cells (*P* < 0.001) [Fig. 3e]. Cells exposed to iron while in 3D culture formed significantly fewer (***P < 0.0001) [Fig. 3e] and smaller (*P = 0.019) [Fig. 3f] colonies than 2D-irradiated cells, with their values matching those of the unirradiated control cells. These results indicate that with a variety of radiation types, 3D culture reduces IR-induced increase in anchorage independent colony growth of HBEC 3KT+*KRas*^v12^+sh*TP53.*

### Protection from transformation by 3D culture persists up to 60 population doublings after exposure to heavy ions

HBECs were propagated in culture for up to four months after iron exposure. 3D-irradiated cells still showed no signs of increased colony formation up to 60 PD. While 2D-irradiated cells formed significantly more colonies than 3D-irradiated cells, the magnitude of these differences diminished with time (**P = 0.003, *P = 0.05) [Supplemental Figure 1]. These data can be interpreted to show that transformation occurs immediately or shortly following IR—there is no long-term latent period where cells must undergo selection.

### Transformation of 2D-irradiated cells is due neither to population differences, nor to proliferation/differentiation status

To ensure the 3D culture method itself did not eliminate a subpopulation of cells during the dissociation process, HBECs were grown in 3D on top of Matrigel (as in Fig. 1c), dissociated into a 2D monolayer culture (as in Fig. 1a), and then exposed to gamma IR. These dissociated 3D cells transform at the same frequency as other 2D-irradiated cells (^*^P = 0.05) [Fig. 4a], indicating the importance of conditions at the time of IR. Thus, the 3D culture dissociation process does not alter or select against potentially “primed” cells that may easily transform in the future. This is also reflected in the equivalent numbers of colonies in both unirradiated controls. This also shows HBECs can transition from 3D to 2D without losing their oncogenic potential.

**Figure 5:**
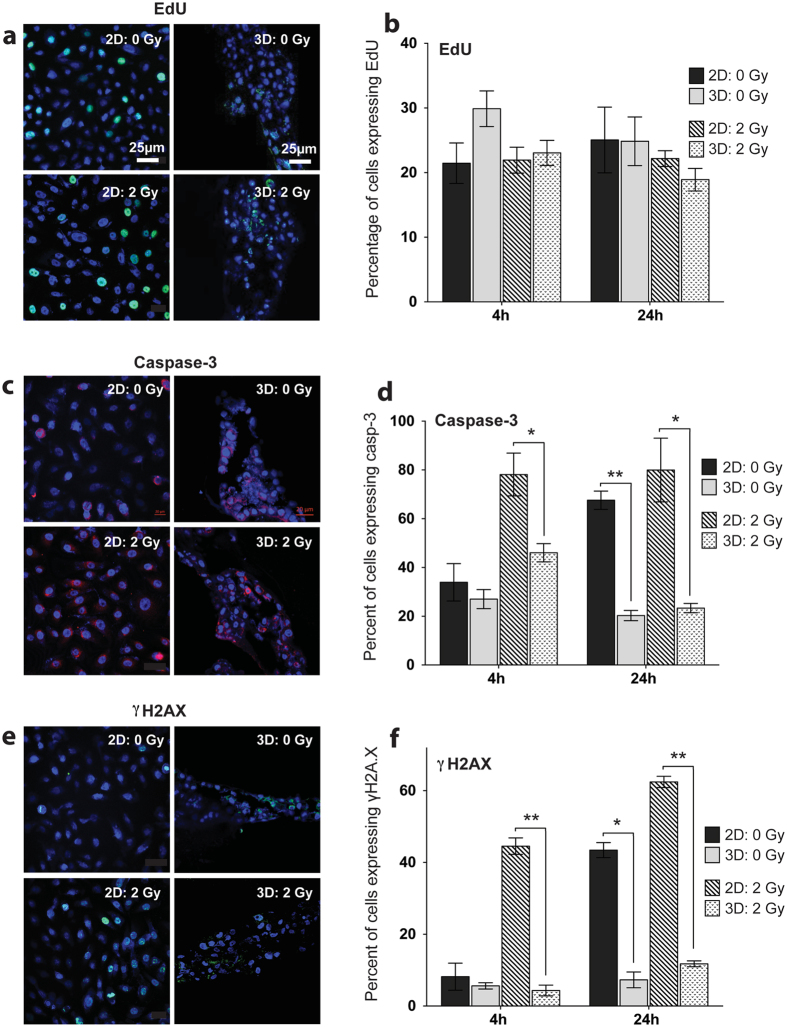
Immunostaining for cellular markers of DNA damage and programmed cell death. Cellular expression of (**a**) EdU, (**c**) cleaved caspase-3, and (**e**) γH2AX in 2D (left) and 3D (right) cultures at 4 h after IR. Percentage of cells expressing (**b**) EdU, (**d**) cleaved caspase-3 (4 h *P = 0.028; 24 h *P = 0.013, **P = 0.0003) (**f**) γH2AX (4 h **P = 0.0001, 24 h **P < 0.0001, ***P = 0.0002) after 2Gy γ irradiation, normalized to total number of cells determined by nuclear DAPI staining. Mean ± SEM.

The majority of cells in grown in 2D with KSFM are in an active stage of the cell cycle, whereas the majority of cells in 3D are differentiated with only 20% actively dividing (shown in Fig. 5e)^21^. To assess whether differentiation media and cell cycle arrest at the time of IR has an effect on transformation, 2D cells were grown to full confluency in 3D differentiation medium (ALI) to temporarily induce cell cycle arrest before IR. These confluent 2D cells still exhibit increased colony formation in soft agar after gamma exposure (*P = 0.05) [Fig. 4b], indicating that neither 3D ALI media nor cell cycle arrest is responsible for reduced transformation seen in 3D cultures.

The presence of fibroblasts may affect 3D transformation, as gene transfer has been documented between fibroblasts and breast cancer cells mixed in culture leading to radioresistance of the latter^22^. HBECs were grown on top of 3D Matrigel without using an underlying fibroblast feeder layer, which results in decreased complexity and branching of the overlying 3D structure^21^. After IR, there is no significant increase in colony formation in 3D cells grown without IMR90s [Fig. 4c]. Other permutations of 3D culture include embedding HBECs in a mixture of 9 parts Matrigel to 1 part ALI (seen in Fig. 1b). After IR, this embedded 3D culture still mitigates the increase in radiation-induced anchorage independent growth seen after gamma IR [Fig. 4c].

### Culture conditions do not affect proliferation rates or cell death after IR exposure

Cells exposed to all three types of IR show similar growth curves soon after IR, regardless of culture conditions [Supplemental Figure 2]. To determine if culture conditions affect the ability of cells to proliferate after IR, we measured DNA synthesis at 4 and 24 hours after IR on whole 3D structures via incorporation of the nucleoside analogue EdU, which was not significantly different between either 2D or 3D, regardless of IR [Fig. 5a,b]. There was also no difference in the survival of cells in the colony formation assay after either γ or iron exposure, regardless of culture conditions [Supplemental Figure 3]. Additionally, there was no increase in number of dead cells cells 90 min after IR in either 2D or 3D conditions [Supplemental Figure 4]. Since cells in both culture conditions grow at similar rates, even after IR, the increased transformation of 2D-irradiated cells cannot be attributed to increased cell death or altered proliferation rates of experimental cell populations.

### Cells irradiated in 3D undergo less apoptosis and DNA damage repair compared to cells irradiated in 2D culture

To visualize induction of apoptosis and expression DNA damage repair, immunohistochemical stains for cleaved caspase-3 and γH2AX were performed either 4h or 24h after 2Gy γ exposure. After IR, cells in 2D had significantly increased staining for caspase-3 at 4 h (*P = 0.028) and 24 h (*P = 0.013), indicating 2D cells undergo more apoptosis compared to cells irradiated in 3D conditions [Fig. 5c,d]. Induction of γH2AX was significantly increased in 2D-irradiated compared to 3D-irradiated cells at both 4 h (**P = 0.0001) and 24 h (**P = 0.0001) [Fig. 5e,f]. Interestingly, unirradiated cells in 2D had significantly increased expression of caspase-3 and γH2AX compared to unirradiated 3D cells (**P = 0.0003; *P = 0.0002) [Fig. 5d,f]. Our data suggest there is decreased DNA damage response in cells grown in 3D culture, and this is supported by the literature^23^,^24^. These results likely reflect the active cellular replication in 2D cultures, requiring upregulated DNA damage and cell cycle checkpoints, leading to higher basal rates of apoptosis and DNA damage repair compared to 3D cells.

### Cells in 2D and 3D upregulate different pathways after IR

**Figure 6:**
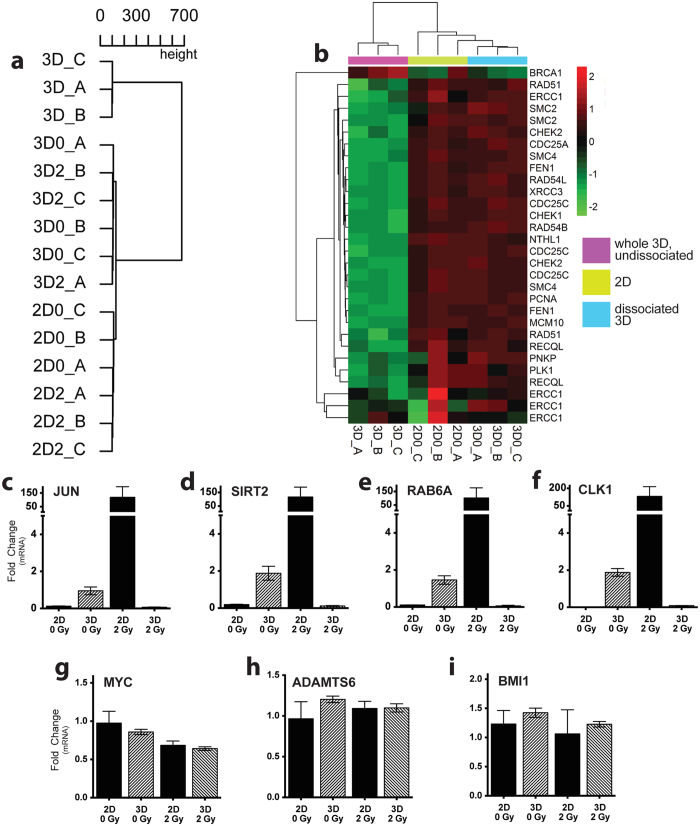
Transcriptional changes between cells in 2D and 3D culture. (**a**) Hierarchical clustering of 2D (2D0, 2D2), whole 3D (“3D”) and dissociated 3D (3D0, 3D2). RNA collected from 3D cells in 3D are vastly different from cells in 2D as well as 3D cells after dissociation. (**b**) Heatmap showing expression of DNA repair genes, which are downregulated in 3D (purple) compared to 2D (green) and dissociated 3D (blue). (**c–i**) qPCR used to validate initial microarray findings shows relative mRNA levels of each gene normalized to housekeeping controls. Mean ± SEM.

Irradiated cells from both 2D and 3D conditions were analyzed for transcriptional differences. Cluster dendrogram revealed a large separation between cells in whole, undissociated 3D culture and all cells grown in 2D grown cells (including 3D cultures after dissociation) [Fig. 6a]. It is no surprise these cells express completely different set of genes, since as discussed in the introduction, they are completely different types of cell culture. It is clear that cells alter their gene expression soon after dissociation from 3D culture, with dissociated 3D cells expressing patterns more similar to that of 2D cells than undissociated 3D cultures [Fig. 6a,b]. A collection of DNA repair genes, described by Asaithamby *et al*.^23^, have decreased expression after IR in 3D culture^23^. We show similar downregulation in whole 3D culture using the same list of DNA repair genes [Fig. 6b].

Using pathway analysis, a subset of genes was chosen based on large fold changes in expression predicted by microarray analysis comparing 2D and dissociated 3D cultures (full lists of genes can be found in the Appendix). The oncogenes Jun and RAB6A were robustly increased in 2D culture after 2Gy gamma IR, as were SIRT2 and CLK1 [Fig. 6c–f]. There were no differences in Myc, ADAMTS6, or BMI1 [Fig. 6g–i].

## Discussion

We show that irradiation of human airway cells in 3D culture, instead of 2D monolayer culture, reduces the frequency of progression toward malignant phenotypes. Importantly, these studies have eliminated potential confounding effects of differing methodologies, including variations in 3D culture, and these all reiterate the reduction in the transformative effects of multiple types of radiation in 3D organotypic culture. Although the mechanism of damage varies depending on the type of radiation, all radiation types can result in cellular damage and mutagenesis. Particle IR such as proton and iron is known to be more efficient at inducing damage thus transforming HBEC 3KT+*KRas*^v12^+sh*TP53* more easily^25^.

Importantly, a comparable number of colonies grow from both 2D and 3D grown cells without IR exposure, indicating transforming cells are not selected out of 3D culture during dissociation, and the transformation rates between 2D and 3D cultures are comparing similar cell populations. Furthermore, cells grown in either 2D or 3D conditions grow comparable proliferation rates determined both by cell growth as well as EdU incorporation [Figs 4 and 5b]. Importantly, 3D cells were assayed for malignant phenotypes after being dissociated from 3D structures, and still they exhibited decreased transformation, even though there is no loss of cells due to differing culture conditions.

Many of our confirmed upregulated genes in 2D irradiated cells (such as Jun and RAB6A) can function as oncogenes, leading to increases in invasive and malignant phenotypes; both Jun and RAB6A are upregulated in multiple types of cancers^26^,^27^. However, SIRT2 has been demonstrated as a tumor suppressor through its role in regulating mitosis and genome integrity^28^. Interestingly, there were no differences in expression of known oncogenes including MYC and BMI1, which has been implicated in proliferative capacity, cell adhesion, and invasion in a variety of cancer types^29^. To confirm relevant genes for radiation response in 3D, these experiments need to be followed up with genetic manipulation studies to determine what specific pathways are responsible for differences in IR-induced transformation of 2D and 3D cells.

These results show that cell culture conditions are fundamental for lung cellular responses to radiation and can affect cancer progression. Since 3D culture is more a biologically representative model of *in vivo* responses, it begs the question if current studies assessing transformation and radiation may be overestimating radiation risks using 2D culture methods. Ideally, expanding the utility of 3D culture can allow for a better understanding of the biological consequences of radiation exposure. Understanding molecular mechanisms that affect radiation-induced DNA damage will be crucial for optimization of cancer therapy and protection of normal tissue.

## Methods and Materials

### 2D Cell Culture

#### Human bronchial epithelial cells

HBECs were obtained from central lung bronchi and immortalized as described previously^30^. Since oncogenically predisposed cells have increased rate of radiation-induced transformation frequency (shown by Din *et al*.)^25^, the HBECs used in this study have been experimentally transformed with expression of *KRas*^V12^ and *TP53* stable knockdown^31^. Immortalized HBEC 3KT+*KRas*^v12^+sh*TP53* were cultured at 37 °C in 5% CO_2_ in keratinocyte serum free medium (KSFM, Gibco) containing 50 μg/mL of bovine pituitary extract and 5 μg/mL of epidermal growth factor on porcine gelatin-coated tissue culture dishes.

#### Primary lung fibroblasts

IMR90 cells, derived from normal lung tissues (ATCC) were cultured in basal media supplemented with 10% calf serum at 37 °C in 5% CO_2_ and 2% O_2_.

### 3D Organotypic Culture

3D cultures were set up using a feeder layer of 250k IMR90 fibroblasts seeded in a 24-well plate 48 h prior to seeding HBECs as previously described^21^,^32^. Undiluted growth factor-reduced, phenol-red free Matrigel (BD Biosciences) was layered on top of the fibroblasts and allowed to solidify. 300k HBEC 3KT+*KRas*^v12^+sh*TP53* were seeded on top of the solidified Matrigel, cultured at the Air Liquid Interface (ALI) at 37 °C in 5% CO_2_ for 5 days and supplemented with media changes containing 10% Matrigel every other day [Fig. 1]^18^.

Within 12 h of IR exposure, 3D structures were dissociated from Matrigel using cell recovery solution (Corning), trypsinized, and cultured as a monolayer alongside 2D. Transformation, proliferation, and colony formation assays were performed on experimental cells after dissociation and passage in 2D culture, usually within three population doublings (PD) [Fig. 1d].

### Radiation

Gamma radiation exposures using a ^137^Cs source at 243.08 cGy/min (Department of Radiation Oncology, UT Southwestern). Charged particle radiation experiments (iron, proton) were performed at the NASA Space Radiation Laboratory (Upton, NY). Cultures were exposed to 0.25 Gy ^56^Fe at 600 MeV/nucleon, or to 0.5, 1, or 2Gy+H at 150 MeV/nucleon.

### Transformation Assays

#### Anchorage Independent Soft Agar

After IR, 8,000 viable cells were suspended and plated in 0.33% Difco Noble agar (BD Biosciences) in KSFM in six replicates in 12-well plates, layered over a 0.5% agar base^25^. The number of macroscopically visible colonies (0.5 mm) was counted 25 days later with imaging at 0.63× using the Zeiss Axiovert 100M and quantification using ImageJ. Each experiment was repeated 4 times.

#### Matrigel Invasion

8,000 viable cells were suspended in KSFM and seeded in the top chamber of 24-well 8-μm pore invasion chambers (BD Biosciences). The bottom chamber was supplemented with 5% calf serum as a chemoattractant. Cells were allowed to migrate for 18 h, then processed per manufacturer’s protocol. Hoechst 33342 stain (Sigma) was used to visualize nuclei of invaded cells at 20× using the Zeiss Axiovert 200M and quantified using ImageJ. Each experiment was repeated 3 times.

### Proliferation Assays

To assess for variations in cellular proliferation following IR exposure, 4,000 cells were cultured in triplicate in 6-well plates, and then trypsinized and counted every two days using an automated cell counter (Bio-Rad).

### Colony Formation Assay

Cells were seeded in triplicate in 10-cm dishes at clonogenic density (100 cells per dish) for colony formation assays. Ten days later, dishes were stained with a mixture of 6.0% glutaraldehyde and 0.5% crystal violet, and colonies (defined as clusters of >50 cells) were counted.

### Immunofluorescence Staining

Cells were fixed with 4% paraformaldehyde at 4 h or 24 h after gamma exposure. Aggregated budding structures from 3D Matrigel cultures were processed and stained as previously described^21^. Primary antibodies used for immunofluorescence include: cleaved caspase-3 (Cell Signaling #9664), anti-phospho-histone γH2A.X (1:400) (Millipore #DAM1479572). Proliferating cells were marked using the Click-iT Edu Alexa Fluor 488 kit according to manufacturer’s instructions (Invitrogen). Sections were mounted using Prolong Gold Antifade Reagent with DAPI (Life Technologies #P36931) and imaged using a confocal microscope. Each staining was performed on multiple sections in triplicate.

### Live/Dead^®^ Cell Assay

To determine cell viability and cytotoxicity within 4 h of IR exposure (on undissociated, untrypsinized cells), cells were stained with 2 μM calcein AM and 4 μM EthD-1 using the LIVE/DEAD^®^ kit (Invitrogen) as per manufacturer’s instructions.

### Microarray Analysis

RNA from each experimental condition with 2Gy gamma exposure, as well as undissociated 3D structures, was collected using an RNeasy Plus mini kit (Qiagen). Biotin labeled cDNA was prepared using Illumina TotalPrep kit (Ambion), and quality of total RNA and biotinylated cDNA checked using the Experion system (Biorad). HT12v4 Beadchip hybridization was performed following Illumina standard protocol (Ambion). Briefly, 750 ng of biotin labeled cDNA was hybridized to the chip overnight at 58 °C, followed by washing and staining. HT12v4 Beadchips were scanned on the Illumina HiScan scanner and data analyzed with Illumina Beadstudio software.

### Quantitative Reverse Transcription PCR

RNA was extracted from 2D and dissociated 3D cultures using an RNeasy Plus kit (Qiagen), and 1 μg of total RNA was reverse transcribed with the iScript™ first-strand cDNA synthesis kit (BioRad). Following cDNA synthesis, quantitative PCR was set up using SsoFast™ EvaGreen^®^ supermix (Biorad) with optimized cycling conditions for LightCycler 480II (Roche). Based on preliminary analysis of microarray data, the following genes were selected as being differentially up- or downregulated between 2D and 3D conditions: Jun proto-oncogene (JUN), sirtuin 2 (SIRT2), Ras-related GTP binding protein (RAB6A), and CDC-like kinase 1 (CLK1), MYC, ADAM metallopeptidase with thrombospondin type 1 motif 6 (ADAMTS6), and BMI polycomb ring finger oncogene (BMI1). Housekeeping genes used were glucuronidase beta (GUSB), heat shock protein 90 kDa alpha class B member 1 (HSP90AB1), and hypoxanthine phosphoribosyltransferase 1 (HPRT1).

### Statistical Methods

Each two-way comparison was analyzed using student’s t-test to determine significance.

All microarray analyses were performed using R version 3.0.1 and Bioconductor version 2.13^33^,^34^. Data were screened for outliers using the lumi package, and processed using non-parametric background subtraction and median normalization using the MBCB package^35^,^36^.

The detection P-value for the twelve samples, and 16,442 probes (*P* < 0.05) were used in all subsequent analysis [Supplemental Table 1]. Significant gene sets were determined between two groups using t-tests and Benjamini–Hochberg procedure to control the false discovery rate (FDR < 0.05) implemented with the multtest package, with fold change calculated using samr^37^,^38^. For each gene list, up- and down- regulated genes were analyzed separately using Qiagen’s Ingenuity^®^ Pathway Analysis (IPA^®^, www.qiagen.com/ingenuity). The 2D0 vs 3D0 (no irradiation) and 2D2 vs 3D2 (2Gy IR) comparisons were re-analyzed using both limma and the more conservative eBayes statistic with fold change calculation as the difference of log-intensities (Appendix A-B)^39^. Overlapping genes were imported into IPA with up- and down- regulated genes analyzed separately [Supplemental Table 1]. Complete lists of genes with significant expression changes, separated by analysis and comparison, can be found in the appendix. The expression profile for the DNA damage response genes were compared in unirradiated samples, and heatmaps were generated using pheatmap package with dendrograms using average clustering with Euclidean distance for both samples probes^23^,^33^.

## Additional Information

**How to cite this article**: El-Ashmawy, M. *et al*. Organotypic culture in three dimensions prevents radiation-induced transformation in human lung epithelial cells. *Sci. Rep.* **6**, 31669; doi: 10.1038/srep31669 (2016).

## Supplementary Material

Supplementary Information
