# Supplementary material for: Organotypic culture in three dimensions prevents radiation-induced transformation in human lung epithelial cells
Source: Sci Rep. 2016 Aug 19;6:31669. doi: 10.1038/srep31669 (PMC4990973; doi:10.1038/srep31669)
Supplement: Supplementary Information [file srep31669-s1.pdf]

Organotypic culture in three dimensions prevents radiation-induced transformation in human lung epithelial cells.

Mariam El-Ashmawy, Melissa Coquelin, Krishna Luitel, Kimberly Batten, Jerry W. Shay*

Supplemental Figures

Supplemental Table

Supplemental Legends

Appendix

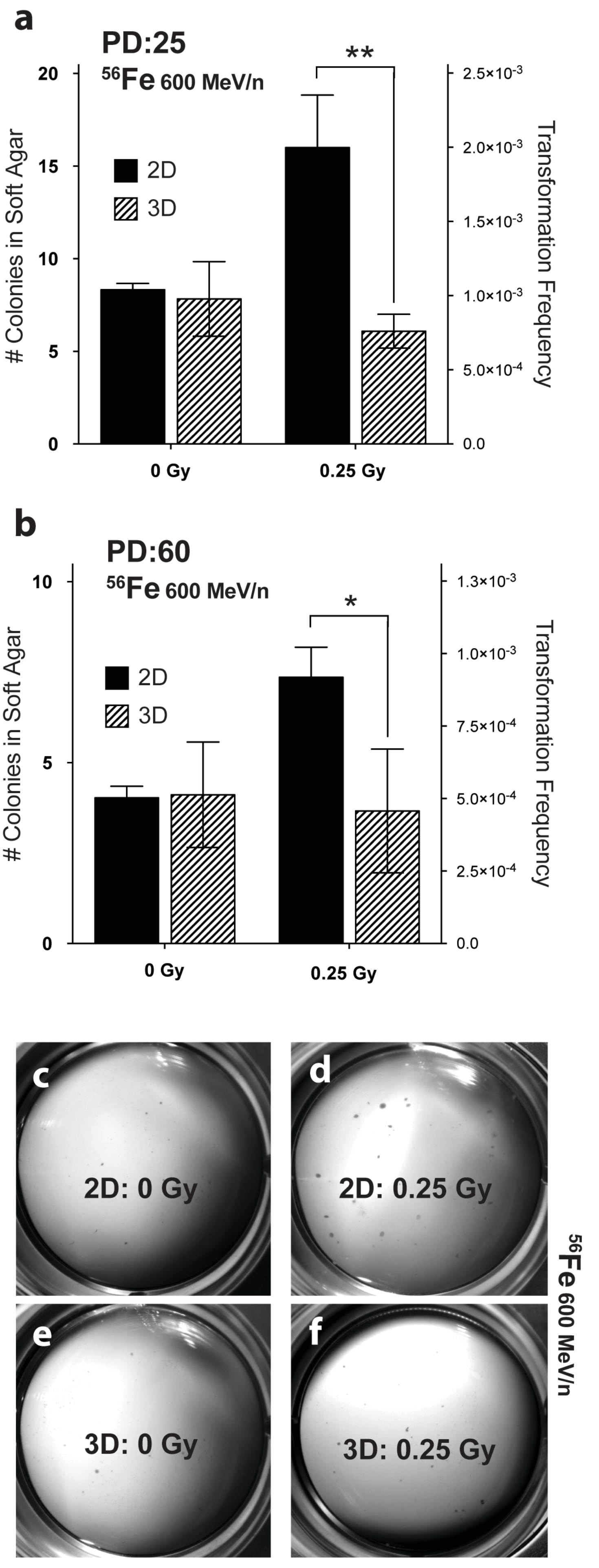

Supplemental Figure 1

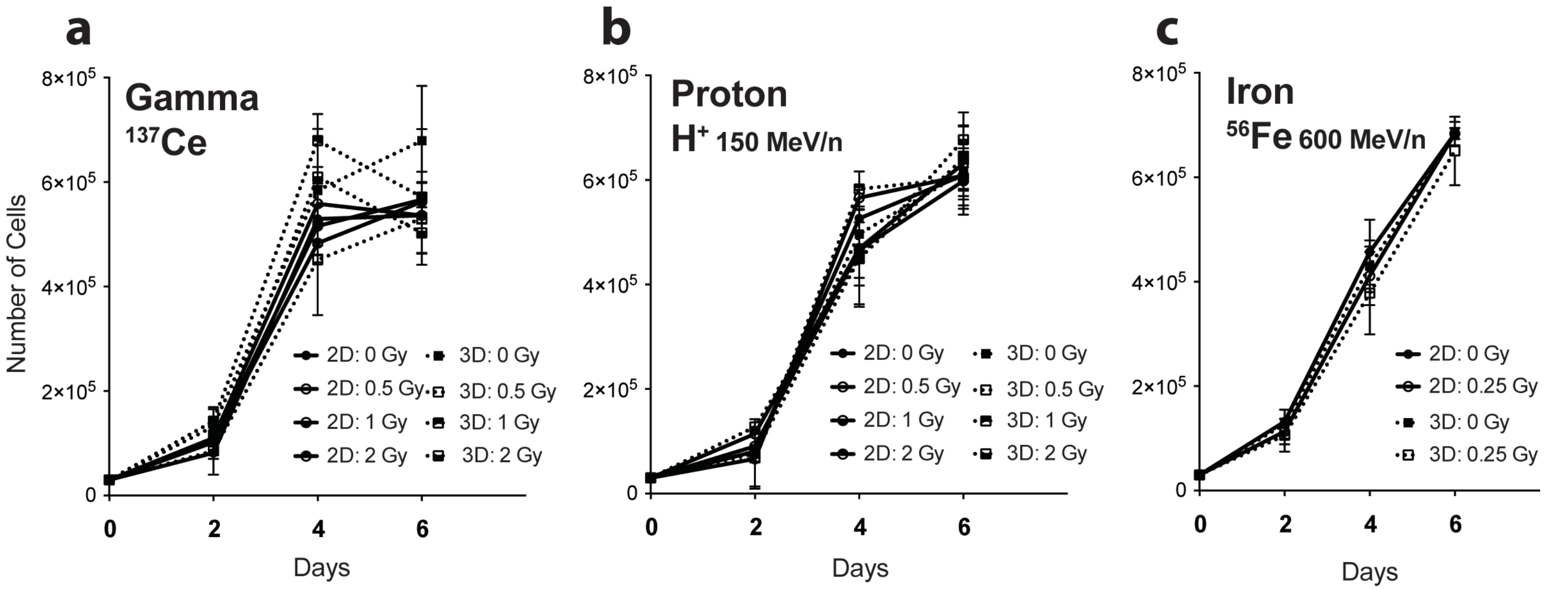

Supplemental Figure 2

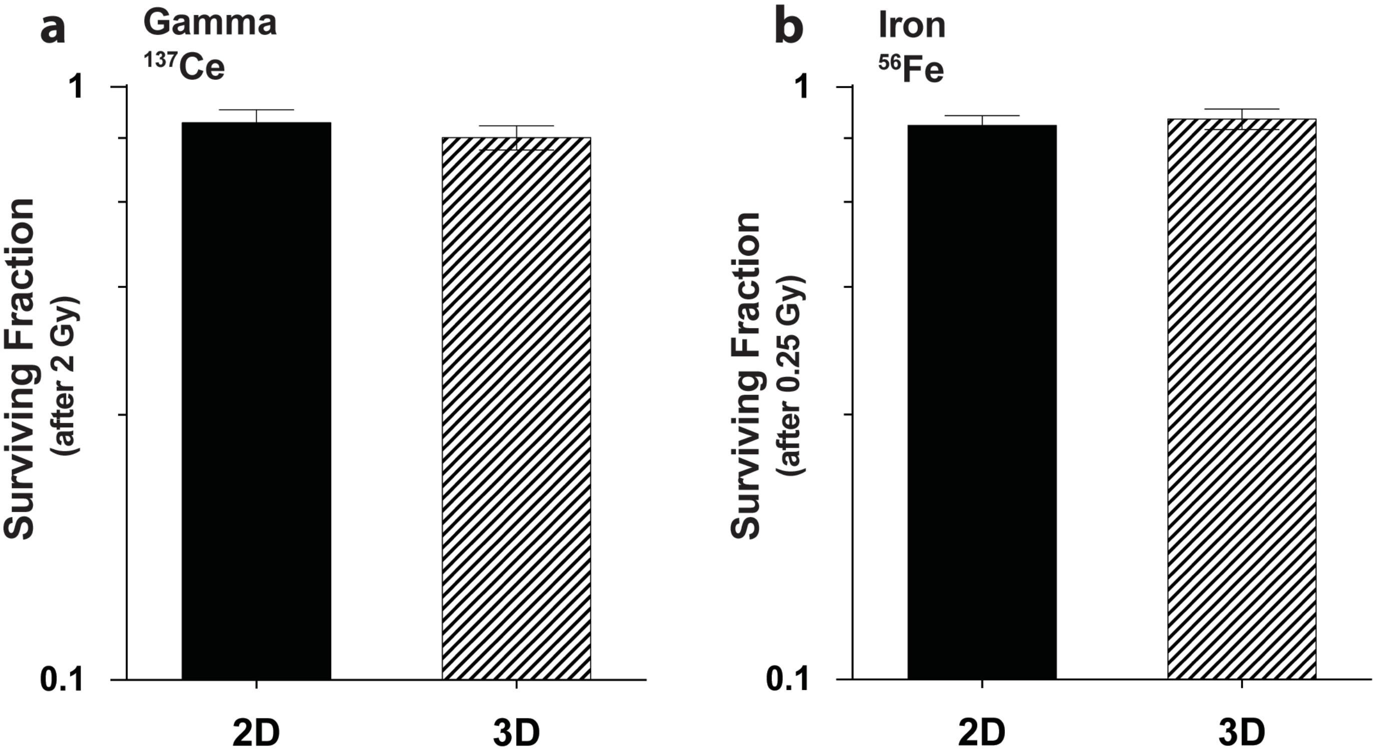

Supplemental Figure 3

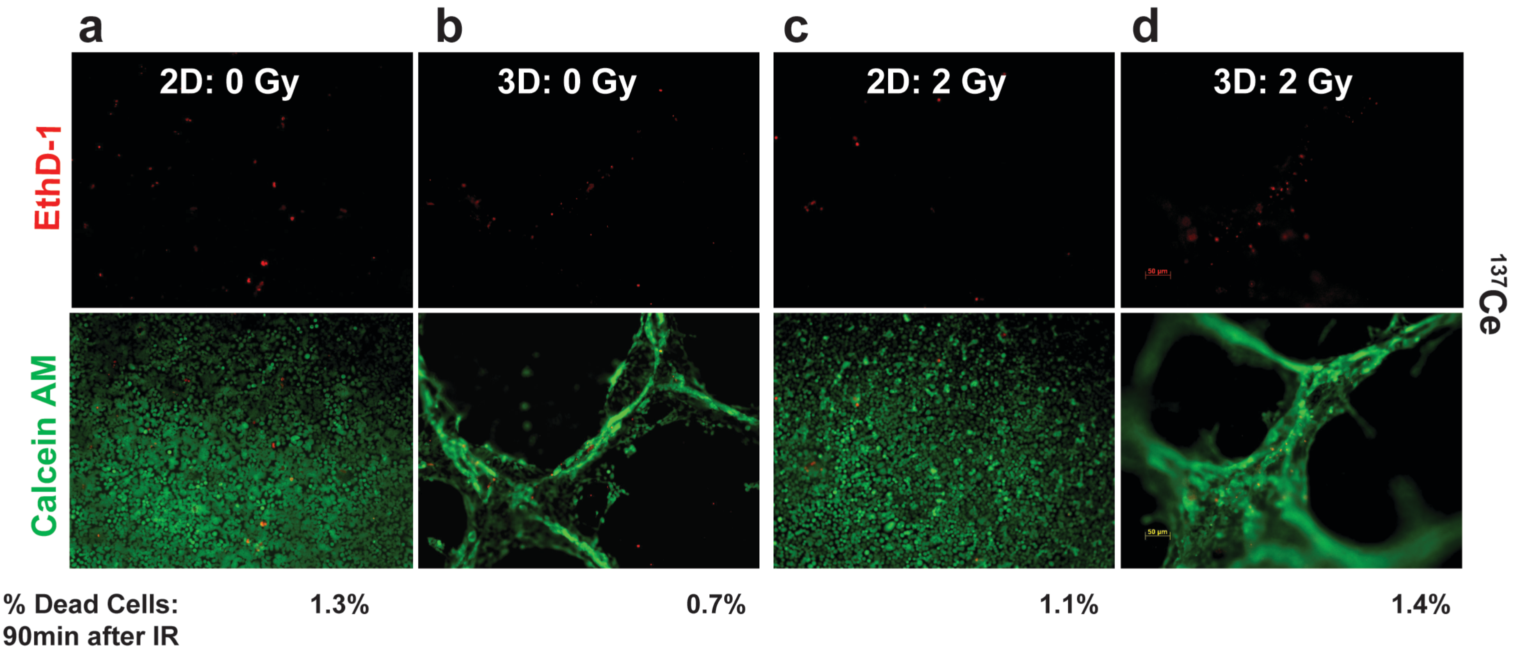

Supplemental Figure 4

Analysis	Significant probes	Up-regulated	Down-regulated
**2D0 v 3D0**	2,301	1,813	488
**2D2 v 3D2**	603	267	336
**2D0 v 2D2**	831	682	149
**3D0 v 3D2**	3,357	2,857	500

	Significant probes w no overlap	Up-regulated	Down-regulated
**Limma 2D0 v 3D0**	533	291	242
**Limma 2D2 v 3D2**	857	513	344

Supplemental Table 1: Number of probes with significant expression changes from each comparison. 2D0: monolayer culture no irradiation; 3D0: organotypic culture no irradiation; 2D2: monolayer culture 2 Gy IR; 3D2: organotypic culture 2 Gy IR.

Supplemental Legends:

Supplemental Figure 1: Soft agar colonies after long-term culture after ^{56}Fe exposure.

Number of colonies after (A) 25 PD and (B) 60 PD after IR shows that 3D-irradiated cells still have no increase in anchorage independent growth when compared to 2D-irradiated cells. *P=0.05, **P=0.003; mean ± SEM. (C-F) Representative images of soft agar colonies. The colonies in panel (D) from 2D with 0.25Gy are the largest and most numerous.

Supplemental Figure 2: Cell growth curves within three population doublings after IR exposure.

Cells irradiated with (A) γ (B) proton, and (C) iron all have similar growth and proliferation patterns, regardless of cell culture conditions at the time of IR. Mean ± SEM.

Supplemental Figure 3: Colony formation assay of cells soon after IR.

There is no difference in the ability of cells to survive and replicate after irradiation. Mean ± SEM of the surviving fraction of cells after (A) 2Gy γ and (B) 0.25Gy iron (600 MeV/n).

Supplemental Figure 4: Dead cells in 2D and 3D cultures 90 minutes after IR.

Staining of γ-irradiated cultures with ethidium-D1 and calcein AM 90 minutes after IR shows no difference in the number of dead (EthD1, red) cells compared to total cells between (A) unirradiated 2D and (B) unirradiated 3D cultures. Irradiated cultures (C) 2D with 2Gy (D) 3D with 2Gy also show no increased numbers of dying cells.

Supplemental Table 1: Number of probes with significant expression changes from each comparison.

2D0: monolayer culture no irradiation; 3D0: organotypic culture no irradiation; 2D2: monolayer culture 2Gy IR; 3D2: organotypic culture 2Gy IR.

Appendices:

Appendix A: Genes with significant expression changes after removal of overlapping probes (limma) comparing 2D versus dissociated 3D HBECs with no irradiation.

Gene Symbol	Log Fold Change
LOC643272	1.026
DBN1	1.007
LOC653107	0.868
ZDHHC13	0.863
MIR221	0.853
CAPRIN2	0.847
WSB1	0.779
MATR3	0.776
MIRLET7D	0.761
ITSN1	0.754
HS.493947	0.739
GJB3	0.725
TAGLN3	0.713
VNN1	0.711
LTBP4	0.706
GAGE4	0.703
SPRY4	0.700
GOLGA8B	0.692
NET1	0.690
DVL1	0.681
CAPRIN2	0.677
ID1	0.673
LOC100133803	0.661
PLAA	0.657
CSF3	0.656
CTPS2	0.653
PCTK1	0.643
CSF3	0.641
EHF	0.629
SNORD94	0.629
PLCG1	0.627
FAM108B1	0.622
BNC1	0.616
RGNEF	0.611
C10ORF46	0.609

Gene Symbol	Log Fold Change
AMPH	0.603
IL1RL1	0.601
EIF3CL	0.593
KIAA0261	0.591
NET1	0.590
CPEB2	0.589
ANGPTL4	0.589
LOC100130623	0.588
HIP1R	0.581
PKP3	0.579
LOC650909	0.579
NAV3	0.575
CD274	0.572
ATRN	0.568
SLC38A1	0.558
HBEGF	0.550
EFTUD1	0.545
NPIP	0.545
HS.551128	0.545
GPR3	0.541
MTSS1	0.540
GTF2IP1	0.539
SERPINB2	0.539
MGC5139	0.536
LOC652388	0.535
KLF10	0.534
SERPINE1	0.533
LOC200030	0.530
COBRA1	0.529
FOS	0.524
LAMB3	0.520
CDK5R1	0.520
AKAP12	0.518
KLF10	0.515
OSMR	0.514

Gene Symbol	Log Fold Change
TRIML2	0.512
HERC3	0.511
SHC1	0.508
LOC100129982	0.502
IL1RL1	0.500
ETS1	0.499
DUSP6	0.499
ILF3	0.497
TGFA	0.494
AP2B1	0.493
ANGPTL4	0.488
AP2B1	0.486
TSC22D2	0.485
SLC25A24	0.480
ACIN1	0.480
SNX30	0.479
CASC4	0.477
SNORA18	0.476
MSN	0.476
SEC24B	0.474
LOC399959	0.473
SS18L1	0.465
RPS6KA3	0.465
TP73L	0.464
SGK1	0.464
SPRY4	0.463
SMTN	0.462
DVL3	0.460
ZCCHC3	0.456
LAMC2	0.453
MOBKL2B	0.452
AMMECR1L	0.452
SLC4A7	0.451
SPTBN1	0.450
RALGAPB	0.449

Gene Symbol	Log Fold Change
LOC92755	0.448
DHX9	0.445
ASAM	0.444
TJP1	0.444
LRP11	0.440
ATP2A2	0.438
ZC3H12A	0.437
ZNF697	0.437
JAK1	0.436
CLIP4	0.431
BAT2	0.430
MATR3	0.428
PTPRM	0.426
MCL1	0.426
SOX7	0.425
XPOT	0.423
HERPUD1	0.423
PHKA2	0.423
TAGLN3	0.422
CAPRIN1	0.422
RYK	0.419
ADIPOR1	0.418
KIAA2010	0.417
BNC1	0.416
HS.558212	0.416
SPAG9	0.416
SMAD3	0.415
ZFC3H1	0.415
TRIB1	0.414
CDKN2B	0.414
SLC39A6	0.411
PABPN1	0.409
SMG7	0.409
LOC652688	0.407
CSRNP2	0.405
SIAH2	0.404
STX6	0.404
CHD7	0.403
FLNB	0.402
ATP5I	0.394
FHOD1	0.394

Gene Symbol	Log Fold Change
KIF5B	0.392
PTPRA	0.392
HK2	0.392
G6PD	0.391
LAMB1	0.388
SGK1	0.387
BCOR	0.385
NRD1	0.384
SH2B3	0.384
C16ORF52	0.383
TRIM5	0.381
ACSS2	0.381
TGM2	0.380
HERPUD1	0.380
CTGF	0.379
ASAP1	0.378
CYB5D1	0.378
NCOR2	0.376
FNBP4	0.376
KDELR3	0.376
ENO2	0.374
GDI1	0.373
BNC1	0.373
CD44	0.371
TNKS1BP1	0.371
LAMC2	0.371
SOX9	0.370
MYEOV	0.368
ASAP2	0.367
KIAA1539	0.367
KPNA6	0.367
SDHA	0.367
INO80C	0.366
MICALL1	0.365
MTMR3	0.365
RERE	0.362
LOC653820	0.362
UBQLN1	0.361
SNRK	0.361
NCOR2	0.361
G3BP2	0.361

Gene Symbol	Log Fold Change
POLM	0.360
SDCBP2	0.360
TINAGL1	0.359
ITGB1	0.358
TRIM44	0.357
CSNK2A1	0.356
RBL2	0.355
PKP1	0.355
ACSS2	0.355
TNFAIP1	0.354
SPIRE1	0.354
DUSP5	0.352
CCND2	0.351
JOSD1	0.350
DDEF2	0.350
MTSS1	0.349
PITPNM1	0.347
TUBB	0.344
CSNK1D	0.344
HMGA1	0.342
PIAS3	0.342
CDCP1	0.341
IL13RA2	0.341
ZNF275	0.341
DGKA	0.340
PTPRE	0.340
ASAP1	0.340
HNRPK	0.339
EXOSC10	0.339
NUP153	0.333
TPX2	0.332
TJP1	0.331
LOC652846	0.324
EIF4B	0.323
IL1A	0.323
LOC728188	0.322
VPS33B	0.322
ARF4	0.321
FNTA	0.321
LAMA3	0.320
LSM12	0.319

**Gene Symbol**	**Log Fold Change**
SURF4	0.316
IQCB1	0.316
MORF4L2	0.314
BAT2D1	0.314
RPS6KA4	0.314
IWS1	0.313
FOSL1	0.312
CREB3L2	0.311
SH3KBP1	0.308
RBM15	0.307
NPC1	0.305
FUBP3	0.303
RAB5C	0.301
CAV2	0.300
DCAF7	0.297
MGEA5	0.297
RAP2A	0.296
ACTB	0.294
MAPKAPK2	0.293
FADS1	0.289
ANKRD57	0.284
GSS	0.282
LOC647000	0.281
IL1B	0.281
GJB3	0.280
UFM1	0.279
SRXN1	0.278
PTMS	0.276
ACTR1B	0.275
COMMD6	0.273
BAIAP2L1	0.271
ERRFI1	0.271
CDK5RAP1	0.268
LARP1	0.268
SLC20A1	0.267
EFHD2	0.263
DERL1	0.261
FKBP1A	0.257
HDGF	0.257
SFN	0.256
LTBR	0.255

**Gene Symbol**	**Log Fold Change**
LOC642489	0.252
DCTD	0.252
PCBP2	0.251
TNFRSF6B	0.250
C19ORF22	0.247
ACTG1	0.243
LAMA3	0.242
CALM1	0.241
RBM12	0.239
SRC	0.238
C11ORF10	0.237
MED24	0.235
SYPL1	0.221
YWHAZ	0.210
LOC641814	0.174
EDF1	-0.191
PRDX5	-0.205
ROBLD3	-0.222
KDELR1	-0.227
TMEM111	-0.228
MRPS22	-0.229
DCTPP1	-0.234
ATP6AP1	-0.236
SF3B5	-0.238
YIF1A	-0.240
MRPS12	-0.244
NDUFS3	-0.245
BUD31	-0.250
PHPT1	-0.250
EFNA1	-0.254
COPS3	-0.257
SF3A2	-0.257
LOC644511	-0.261
COPS5	-0.261
SNRPC	-0.264
LOC649447	-0.265
KRT8	-0.266
COMMD4	-0.268
AK3L1	-0.268
NT5C3	-0.270
ANAPC11	-0.270

**Gene Symbol**	**Log Fold Change**
SEPX1	-0.273
C8ORF55	-0.273
UROS	-0.274
SUMF2	-0.275
LOC100128196	-0.278
RFXANK	-0.278
NMRAL1	-0.279
SNRPN	-0.279
MRPS26	-0.280
LOC390557	-0.282
CIAPIN1	-0.283
HSBP1	-0.284
NOP56	-0.289
UBXN2A	-0.289
HINT2	-0.292
RPP40	-0.294
SDHAF2	-0.295
TRIAP1	-0.295
NIP7	-0.297
SLC2A1	-0.298
BOLA3	-0.299
SDF2	-0.299
ATL3	-0.299
SKP2	-0.301
DNAJC8	-0.301
MGMT	-0.301
DCXR	-0.309
BCAS4	-0.309
H2AFJ	-0.310
RNASEH2A	-0.310
HOXB7	-0.312
C7ORF59	-0.312
LOC643856	-0.312
LOC648390	-0.312
SIL1	-0.313
TMEM160	-0.313
RHBDD2	-0.313
C10ORF116	-0.314
NDUFA9	-0.320
DNTTIP1	-0.321
EDF1	-0.323

Gene Symbol	Log Fold Change
ORC5L	-0.324
TEX264	-0.325
UBE2L6	-0.325
SNRPB	-0.325
ZYX	-0.326
C20ORF27	-0.326
ACTR10	-0.327
NDUFS7	-0.328
TSNAX	-0.328
LYRM4	-0.328
PEX16	-0.329
DHRS7B	-0.329
STAP2	-0.329
TPD52L1	-0.332
PQBP1	-0.333
LOC100130562	-0.333
CD320	-0.333
CMBL	-0.333
C7ORF49	-0.339
PDIA6	-0.341
UBXN4	-0.342
PMVK	-0.343
KIAA0114	-0.344
KLHDC4	-0.344
S100A9	-0.347
RHBDD2	-0.348
GNPTG	-0.350
RPL14L	-0.350
UBAC2	-0.351
TP53I13	-0.352
BOLA3	-0.352
MRPS18B	-0.355
BIRC5	-0.356
NR2C2AP	-0.356
ARL6IP4	-0.356
DGCR6	-0.358
CHCHD3	-0.360
TMSL3	-0.362
TARBP2	-0.365
COMT	-0.366
C7ORF10	-0.367

Gene Symbol	Log Fold Change
CDC42	-0.368
TINF2	-0.368
ACOT9	-0.368
LOC643438	-0.370
RAD51AP1	-0.371
PLLP	-0.372
DHCR24	-0.374
LOC729992	-0.376
BCL7B	-0.376
COMMD9	-0.378
LOC729774	-0.378
ZNHIT1	-0.378
MRPL27	-0.379
ISG15	-0.379
LOC388556	-0.379
ITPA	-0.383
LOC387703	-0.383
NUDT16L1	-0.385
C16ORF13	-0.385
SCARNA10	-0.388
MRPS11	-0.388
ATP6V0E2	-0.389
SNAP47	-0.389
ARD1A	-0.391
CWF19L1	-0.392
COPE	-0.394
LOC91561	-0.397
LEPROT	-0.399
UBIAD1	-0.399
NT5C3	-0.402
WBP1	-0.404
KLHDC8B	-0.404
KCNN4	-0.405
HS.535360	-0.409
PSMB8	-0.412
GANAB	-0.418
ASPSCR1	-0.421
PAQR7	-0.422
ABHD14A	-0.424
SNRPC	-0.427
NINJ1	-0.431

Gene Symbol	Log Fold Change
CBR1	-0.436
ANKRD22	-0.436
HAX1	-0.438
MSL3L1	-0.439
SLC22A18AS	-0.444
GCHFR	-0.444
RPAP2	-0.445
SLC35F2	-0.449
TMBIM1	-0.450
TCP1	-0.455
ARID4B	-0.456
LOC100131471	-0.461
SAR1A	-0.462
C7ORF68	-0.464
C14ORF142	-0.464
TCF7	-0.466
XAF1	-0.467
PINX1	-0.467
C4ORF48	-0.471
DDX49	-0.472
HIST2H2AA3	-0.474
DGCR6L	-0.475
CHP	-0.476
C9ORF23	-0.478
ECE2	-0.480
RER1	-0.481
LOC644214	-0.481
ECSIT	-0.486
OIP5	-0.487
LOC100128056	-0.487
DSCC1	-0.489
TTC8	-0.491
LOC100130071	-0.493
AYP1P1	-0.498
LOC100129566	-0.499
RMI1	-0.502
CCDC101	-0.502
DENND2A	-0.504
PRNPIP	-0.506
FAM114A2	-0.506
GSTO2	-0.511

Gene Symbol	Log Fold Change
TAF5L	-0.518
C2ORF79	-0.521
KRT15	-0.522
MANBAL	-0.522
FBXO32	-0.522
NCRNA00094	-0.524
ZC3H5	-0.538
C11ORF83	-0.539
HAX1	-0.541
AFMID	-0.543
PYCARD	-0.545
CARD14	-0.547
SF3B3	-0.553
DBP	-0.556
LOC100128627	-0.567
ORMDL2	-0.569
LOC100129297	-0.575
RGL1	-0.588
HIST2H2AA4	-0.588
C22ORF27	-0.603
LOC729157	-0.604
GOLGA7	-0.607
SGCB	-0.622
PXMP4	-0.623

Gene Symbol	Log Fold Change
IFIT3	-0.635
IL7R	-0.665
HS.543956	-0.667
PRPF40A	-0.668
MAP1D	-0.669
HS.542027	-0.672
DENND2D	-0.685
FBXO16	-0.691
LOC100133565	-0.700
HS.580169	-0.700
HS.542579	-0.701
LOC100132428	-0.730
HS.325396	-0.741
TFF3	-0.750
FBXO32	-0.770
IFIT1	-0.778
LOC100133692	-0.838
LOC100131970	-0.842
TSPAN1	-0.877
B3GNT1	-0.906
SOX2	-0.928
SOX2	-1.073

Appendix B: Genes with significant expression changes after removal of overlapping probes (limma) comparing 2D versus dissociated 3D HBECs after 2 Gy.

Gene Symbol	Log Fold Change
TXNL1	1.167
LOC441550	1.027
HSPE1	1.014
SNORA32	1.000
LOC654350	0.978
SNORD80	0.946
PDCD10	0.943
C3ORF14	0.940
LOC440063	0.933
RNF39	0.917
LOC646949	0.911
GNG10	0.889
TSC22D1	0.880
FABP5	0.860
MRPL47	0.842
LOC441073	0.835
AGR2	0.833
ZNF280D	0.832
CROP	0.832
LOC100129657	0.830
ACPT	0.826
ATF4	0.822
LOC729009	0.817
SNORD49A	0.815
CLK1	0.813
LOC100128291	0.799
PODXL2	0.787
ZNF22	0.778
B3GNT5	0.772
KRCC1	0.763
LOC388122	0.761
FZD6	0.760
CSTF3	0.760
LOC641848	0.753
TCEA1	0.753
LOC643911	0.753
GOLT1B	0.749

Gene Symbol	Log Fold Change
LOC652541	0.747
RPL9	0.735
TXNDC9	0.734
LOC100131672	0.733
PMS2L2	0.724
GOLGA6B	0.721
DCAF6	0.718
LOC729236	0.715
TP53INP1	0.715
LOC648822	0.706
LOC390183	0.704
LOC649555	0.698
LOC100130932	0.697
FLJ22639	0.693
HS.363510	0.692
C11ORF73	0.692
LOC440991	0.692
ARL17P1	0.691
LSM6	0.687
LOC100129237	0.687
HS.314414	0.686
RNPC3	0.684
NIPAL2	0.683
RTN4	0.680
LOC730029	0.680
LOC100128060	0.675
UFM1	0.674
LOC653631	0.671
CENPQ	0.671
ARRDC4	0.670
ANKRA2	0.669
BUB3	0.669
SCG5	0.669
LOC100131205	0.666
LOC100128440	0.662
ZNF404	0.660
LOC728602	0.659

Gene Symbol	Log Fold Change
RTN3	0.657
LOC100128337	0.656
BZW1	0.655
LOC100129067	0.650
C9ORF85	0.648
LOC729686	0.645
ALS2CR8	0.644
LOC645968	0.644
C6ORF120	0.642
HS.405877	0.642
LOC440145	0.641
CXADR	0.638
LOC100132547	0.635
PERP	0.635
TRA1P2	0.634
LOC100128060	0.633
LOC391370	0.633
C9ORF163	0.630
SFRS11	0.630
LOC727821	0.629
FLJ43681	0.628
CDC2	0.628
TMEM167A	0.627
CCDC90B	0.627
FAM162A	0.623
NNMT	0.623
LOC100129759	0.622
SLPI	0.622
NDUFB9	0.622
TCN1	0.613
FBXO38	0.612
KLRC3	0.610
P704P	0.610
CHPT1	0.609
C6ORF173	0.609
LOC392285	0.608
ASPH	0.608

Gene Symbol	Log Fold Change
TFPI	0.606
LOC100128689	0.606
NAP1L1	0.606
SOAT1	0.604
APIP	0.604
LOC100134504	0.603
HLA-F	0.602
PMS2	0.602
GAGE12F	0.599
LOC100129742	0.599
LOC642502	0.597
LOC100130154	0.595
LOC100131526	0.593
RPL23	0.593
ISCA1	0.592
NACAP1	0.587
ACAT2	0.587
STAT5B	0.587
LOC390735	0.586
ODF2L	0.585
GTPBP2	0.584
LOC341965	0.583
LOC641768	0.581
C20ORF108	0.580
COMMD10	0.580
LOC729208	0.579
C1QL1	0.578
PTGES3	0.578
LOC644615	0.578
LOC730052	0.577
LOC100132673	0.577
LOC653773	0.574
POLR2J3	0.574
SFRS18	0.574
RBM7	0.573
MRPL48	0.571
MCTS1	0.571
LOC286512	0.570
C7ORF28B	0.568
LOC649946	0.567
SCD5	0.564

Gene Symbol	Log Fold Change
PPP4R4	0.562
HNRNPA2B1	0.560
SNORD36A	0.556
DDX17	0.555
DNAJC25-GNG10	0.549
LOC100133277	0.549
OSTC	0.547
LOC100131323	0.545
C2ORF76	0.544
TSPAN13	0.541
MGC12965	0.540
EIF3M	0.538
LOC727865	0.538
LOC441484	0.538
LOC729255	0.538
LOC728484	0.536
ELF3	0.535
YWHAG	0.534
HS.569175	0.534
RRAS2	0.533
LOC100132199	0.531
LOC642989	0.531
LOC645630	0.530
AIG1	0.527
MYC	0.525
UBLCP1	0.524
LOC727984	0.522
LOC100128086	0.520
LOC645157	0.520
LOC729342	0.519
LOC730278	0.518
PMEPA1	0.517
LOC100132139	0.516
LOC729500	0.514
LOC442232	0.513
MND1	0.511
LOC728026	0.510
FAM92A1	0.510
ZNHIT3	0.505
LOC730255	0.505

Gene Symbol	Log Fold Change
SUB1	0.499
LOC644877	0.499
PNN	0.498
LOC653702	0.497
LOC100131672	0.497
AHSA2	0.497
LOC158160	0.496
NCRNA00081	0.495
LOC100129866	0.495
HNRPA1L-2	0.494
LOC641849	0.493
ATAD1	0.492
LOC100129685	0.492
TCEAL8	0.491
SF3B14	0.491
ALDH3B2	0.490
LOC100132992	0.488
EAPP	0.488
ITGB3BP	0.487
LOC100130892	0.487
LOC646527	0.485
MBIP	0.484
C12ORF60	0.483
VKORC1	0.481
RPL7	0.479
CSTF3	0.478
LOC391532	0.477
ETS1	0.475
HAT1	0.474
LOC100132918	0.474
LOC653566	0.473
LOC648210	0.472
COX7B	0.471
LOC729500	0.471
CPOX	0.470
ZFAND6	0.470
BET1	0.468
LOC401537	0.468
NOP56	0.467
C6ORF160	0.466
HNRPC	0.466

Gene Symbol	Log Fold Change
LOC391833	0.464
LOC100131609	0.464
FKBP3	0.461
STX7	0.460
SNRPN	0.459
C4ORF41	0.459
C5ORF44	0.458
ARMCX6	0.458
CDC42SE2	0.456
SMNDC1	0.455
LOC647037	0.455
SIP1	0.454
PPP2R3C	0.454
CD58	0.453
FABP5L2	0.453
C13ORF27	0.452
LOC653071	0.452
FTHL12	0.451
LOC493869	0.450
LOC645691	0.449
HIGD1A	0.449
HIF1A	0.446
MMP28	0.446
LOC653778	0.446
LOC100132291	0.443
IFT52	0.443
LOC389156	0.442
PCNA	0.441
C8ORF59	0.441
LOC646785	0.440
LOC100132457	0.438
CCT6P1	0.437
LOC439953	0.437
SCOC	0.437
PRKAB2	0.437
LOC100129086	0.436
LOC388076	0.436
ARL4A	0.435
SFRS12	0.433
UQCRH	0.430
PRKAR1A	0.429

Gene Symbol	Log Fold Change
SYTL1	0.427
LOC645693	0.426
LOC389787	0.426
ATP5C1	0.426
LOC100133012	0.422
LOC100132863	0.421
FRG1	0.420
TCP1	0.420
B2M	0.419
HSPC157	0.418
LOC653147	0.418
LOC729646	0.418
LOC401640	0.417
LOC440595	0.416
POP4	0.416
C18ORF10	0.415
CNIH4	0.415
LOC100129118	0.414
LIMCH1	0.414
EI24	0.412
MRPS33	0.411
SAR1B	0.411
LOC728732	0.411
COMMD10	0.410
UNC119B	0.410
LOC653658	0.409
ZNF83	0.408
LOC647030	0.408
GNG10	0.408
LOC100131261	0.407
HMMR	0.405
ALG5	0.405
CMPK1	0.405
NAE1	0.404
CCDC104	0.404
DYNLRB1	0.403
P4HA1	0.403
ATF4	0.403
LOC644029	0.403
40800.000	0.402
LOC728244	0.400

Gene Symbol	Log Fold Change
LOC440928	0.399
RPS26L	0.397
LOC654244	0.397
C1ORF41	0.396
ABCG1	0.396
LOC389156	0.394
NIPSNAP3A	0.392
LOC642975	0.391
PSMA3	0.390
LOC645138	0.389
F3	0.388
LOC731640	0.388
CD9	0.387
LOC100129599	0.386
LOC647150	0.385
TIAL1	0.384
MTPN	0.384
EEF1AL7	0.384
SLC35D2	0.383
LOC100132795	0.382
LOC727865	0.382
LOC730324	0.380
PDIA3P	0.379
LOC651149	0.379
PTTG3P	0.378
CRIPT	0.376
LOC100130511	0.376
HNRPDL	0.375
UBE2Q2	0.375
RPL39L	0.374
PGAM4	0.374
SLC25A40	0.373
LOC389662	0.372
MRPS33	0.371
DBI	0.370
LOC440731	0.370
TSC22D3	0.370
MED7	0.369
KLHDC2	0.369
TCEAL8	0.368
PPA2	0.367

Gene Symbol	Log Fold Change
LOC100131387	0.367
LOC647081	0.365
NIPAL4	0.365
ABHD3	0.364
TMEM189-UBE2V1	0.363
LOC100131905	0.363
SIP1	0.361
LOC644790	0.361
TSC22D1	0.361
SUMF2	0.360
DSTN	0.360
C9ORF46	0.359
NDUFA4	0.359
CHMP5	0.358
C14ORF109	0.357
NUP54	0.355
LOC644799	0.355
YEATS4	0.355
LOC645715	0.354
NDUFAB1	0.353
ACYP2	0.352
LOC642590	0.352
ZC3H15	0.351
IDI1	0.351
RAP2C	0.351
LOC100127918	0.350
CYCSL1	0.350
STARD3NL	0.347
TUSC1	0.347
LOC641844	0.346
TXNDC5	0.346
OSTC	0.345
C8ORF40	0.344
C3ORF23	0.344
LOC389672	0.343
PSMA6	0.338
CITED4	0.338
SEC11C	0.338
LOC645094	0.338
MGC87895	0.338

Gene Symbol	Log Fold Change
STEAP1	0.335
MAP2K1IP1	0.335
LYRM5	0.334
LOC387825	0.334
LOC100128410	0.334
LOC220433	0.333
C6ORF115	0.333
SPTLC1	0.333
SELK	0.332
MAP7	0.331
MRPL42	0.330
LOC728672	0.327
RBX1	0.327
GGH	0.327
SNHG5	0.324
CSTA	0.321
LOC728739	0.321
CKMT1B	0.320
HS.363526	0.320
ATP1B3	0.320
B2M	0.319
LOC651894	0.317
RYK	0.317
MTX2	0.316
LOC100128266	0.315
LOC151579	0.315
TIPRL	0.315
CLDND1	0.315
CCDC23	0.315
RPL13L	0.314
LOC100130308	0.314
CHMP5	0.312
C17ORF95	0.311
LOC442454	0.310
LMF2	0.310
LOC728782	0.308
TMEM219	0.307
RPS27L	0.307
LOC388654	0.306
GTF2H5	0.304
F2RL1	0.303

Gene Symbol	Log Fold Change
C14ORF109	0.302
LOC646849	0.302
PSMA3	0.300
LOC729362	0.300
ANKRA2	0.300
UBE2E3	0.300
STX8	0.299
TGIF1	0.297
CRTAP	0.296
PPIA	0.296
MGST1	0.296
LOC124512	0.294
ZC3H11B	0.292
PIGF	0.291
RAB5A	0.291
CNIH	0.290
TRAK2	0.290
LOC390354	0.290
DYNLT1	0.288
ZBED5	0.288
TPST2	0.288
TMEM49	0.287
LOC391126	0.287
LOC391656	0.286
TGIF1	0.286
UNC50	0.283
LOC729926	0.281
COPS8	0.279
MGST2	0.276
ANLN	0.275
RAB9A	0.275
YWHAE	0.274
UBE2V2	0.272
LOC401397	0.272
CDKN3	0.271
LOC100128936	0.269
C14ORF166	0.268
TMEM14B	0.266
MRPL13	0.265
LOC646819	0.263
ATP5F1	0.261

Gene Symbol	Log Fold Change
LOC653566	0.258
LEPROTL1	0.258
PDCD10	0.257
TSC22D1	0.255
GGCT	0.253
C11ORF1	0.252
PFN2	0.248
FAM108C1	0.246
LOC441073	0.241
UBE2A	0.240
LOC100131940	0.230
NDUFS4	0.228
TOMM20	0.228
VPS29	0.225
COX7A2	0.222
KDELR2	0.222
MANBAL	0.217
RPS24	0.216
RPLP0	0.214
TRMT5	0.213
S100A11	0.212
HBXIP	0.212
CALM2	0.194
GTPBP4	-0.199
MRPL37	-0.215
PLSCR3	-0.216
ATIC	-0.219
PIN1	-0.221
PHF5A	-0.221
SLC16A3	-0.228
CUTA	-0.229
SERINC3	-0.229
LOC401115	-0.229
PLOD2	-0.232
SEC13	-0.233
RASIP1	-0.234
PARL	-0.234
HAS3	-0.235
BCKDK	-0.235
MAPKAPK3	-0.237
LOC389168	-0.241

Gene Symbol	Log Fold Change
LAD1	-0.244
CAMK2N1	-0.245
MKNK2	-0.248
VIL2	-0.249
PSMD2	-0.252
KRT18P13	-0.252
MAPK13	-0.254
NT5DC3	-0.255
APEX2	-0.258
PLOD3	-0.259
RNH1	-0.266
RBM10	-0.267
EWSR1	-0.268
CCDC86	-0.268
NFKBIA	-0.271
SNX27	-0.274
CDK6	-0.275
HPS6	-0.278
ACTR1A	-0.283
ASH2L	-0.283
GRSF1	-0.283
POLR1E	-0.285
CCND3	-0.286
MUL1	-0.286
PTBP1	-0.288
PRPF4	-0.290
HSPH1	-0.290
DNMT1	-0.291
SRRM1	-0.291
PIK3R2	-0.291
BRMS1	-0.292
ADD1	-0.293
LYAR	-0.294
CASP2	-0.294
LRRFIP2	-0.294
PRDM4	-0.297
PTPLAD1	-0.298
PLEKHB2	-0.298
ZNF787	-0.299
EIF6	-0.299
MTHFS	-0.299

Gene Symbol	Log Fold Change
NUDC	-0.302
ACTN1	-0.303
RANGAP1	-0.303
EIF4A1	-0.303
PAK1IP1	-0.304
BANP	-0.305
NFKB1	-0.305
NOTCH1	-0.306
E4F1	-0.306
METTL13	-0.307
TAF15	-0.309
RHPN2	-0.309
RNMT	-0.310
XPO4	-0.310
AKIRIN1	-0.312
RBM28	-0.312
MCM7	-0.313
HS.370359	-0.315
DDRGK1	-0.315
CCNY	-0.316
FAM46B	-0.317
NTHL1	-0.317
PPM1G	-0.319
FKBP4	-0.320
CTSB	-0.323
RBM14	-0.324
CDC20	-0.324
MACF1	-0.324
HS.27048	-0.328
FBXO21	-0.328
NVL	-0.328
XRCC6	-0.330
TNPO3	-0.330
LOC340260	-0.331
CCDC51	-0.331
ZBTB43	-0.332
GSK3B	-0.333
UGCG	-0.333
GEMIN4	-0.334
FLJ10374	-0.335
PARP4	-0.337

**Gene Symbol**	**Log Fold Change**
C11ORF2	-0.339
WWP2	-0.339
RAI14	-0.340
FEN1	-0.340
SMARCA4	-0.343
PARP1	-0.343
NOL6	-0.344
SYNJ2BP	-0.344
PKP4	-0.344
COL17A1	-0.348
ZC3HAV1	-0.348
FASTKD5	-0.350
HNRPM	-0.352
YY1	-0.352
SPNS1	-0.353
C16ORF35	-0.353
ISG20L2	-0.354
FRYL	-0.355
EIF4G1	-0.356
PCNX	-0.356
LRWD1	-0.358
COBLL1	-0.358
PTPRF	-0.358
RNF121	-0.360
ATG10	-0.362
COG2	-0.363
C1ORF163	-0.363
MYO5C	-0.364
PLEKHA1	-0.364
DDX27	-0.366
LAS1L	-0.366
CCNF	-0.366
NCLN	-0.366
DNM1L	-0.366
NFIB	-0.367
EIF2AK4	-0.368
CFLAR	-0.368
USP14	-0.369
XPO5	-0.370
C1ORF71	-0.371
RPL8	-0.372

**Gene Symbol**	**Log Fold Change**
GNB5	-0.377
PAK4	-0.379
PDSS2	-0.380
PDPK1	-0.383
NUP62	-0.384
UBN1	-0.384
SLC9A1	-0.384
LOC723972	-0.385
CEP350	-0.386
EVI5	-0.387
COL4A1	-0.387
R3HCC1	-0.388
SLC20A2	-0.389
ABCF1	-0.391
SQSTM1	-0.392
FBXW4	-0.392
ZNF593	-0.392
BBX	-0.393
KIAA0355	-0.394
ATF5	-0.394
AHNAK	-0.396
TYW1B	-0.397
DOCK5	-0.399
SLC25A22	-0.402
IL27RA	-0.403
AHNAK2	-0.406
LOC100008588	-0.407
SNTB2	-0.407
PCDH7	-0.408
MED16	-0.408
LOC729535	-0.408
KAT5	-0.408
TCEB3	-0.409
PCYOX1	-0.414
FICD	-0.414
DNM1L	-0.414
DDX19A	-0.415
NUCKS1	-0.419
INO80D	-0.421
MBP	-0.423
PLEKHF1	-0.424

**Gene Symbol**	**Log Fold Change**
PRKDC	-0.425
SASH1	-0.426
EP400	-0.428
NBPF10	-0.428
ASCC2	-0.429
C21ORF57	-0.431
HNRNPL	-0.432
ATRIP	-0.432
TNFRSF10A	-0.433
LOC730316	-0.433
SDSL	-0.435
LOC644422	-0.437
SNTB2	-0.437
CCDC102A	-0.438
FAT1	-0.440
USP9X	-0.440
XPNPEP3	-0.441
FAT1	-0.444
C17ORF53	-0.444
CHD8	-0.445
PIAS4	-0.445
CRTC3	-0.448
GCN1L1	-0.449
ZNF142	-0.450
UBR4	-0.455
NACC2	-0.455
TDG	-0.458
TRIM13	-0.460
GMPPB	-0.460
ASXL2	-0.461
SBF1	-0.464
PXMP4	-0.466
DHX37	-0.467
DNAJB12	-0.468
SF3B1	-0.469
CDT1	-0.472
L2HGDH	-0.479
LOC653103	-0.482
APCDD1L	-0.483
ZZEF1	-0.484
AP1B1	-0.487

Gene Symbol	Log Fold Change
DFFA	-0.488
RASAL2	-0.488
BANP	-0.492
TLN1	-0.494
C14ORF102	-0.496
UBE2O	-0.498
UPF1	-0.501
USP24	-0.504
ITPRIPL2	-0.505
AKAP13	-0.505
TNPO1	-0.506
C21ORF70	-0.516
JARID1A	-0.519
FEM1B	-0.526
NAT10	-0.529
TRRAP	-0.533
DYNC1H1	-0.535
ZNF148	-0.538
DYRK2	-0.539
RNF113A	-0.540
SCAMP3	-0.544
PAPD5	-0.545
LOC645233	-0.547
KCTD12	-0.550
HIRIP3	-0.551
IGF2R	-0.553
EIF2C2	-0.559
CHAF1A	-0.559
EHD1	-0.561
COL4A3BP	-0.563
POLR3A	-0.566
CCDC21	-0.566
FBN2	-0.569
FYTTD1	-0.573
PRKAR2A	-0.575
LIMS1	-0.589
LOC644422	-0.590
SNHG9	-0.595
DIS3L	-0.596
**Gene Symbol**	**Log Fold**
	**Change**
LOC100134098	-0.603
HNRNPUL2	-0.603
HS.482960	-0.603
PDP2	-0.603
HM13	-0.604
RFC2	-0.604
MYCBP2	-0.606
RRBP1	-0.607
ZBTB40	-0.609
RAB35	-0.611
ZNF320	-0.615
TNRC6B	-0.622
INCENP	-0.622
CUEDC1	-0.625
B3GNTL1	-0.626
SRGAP1	-0.631
ZNF594	-0.633
LOC100129269	-0.634
LRAP	-0.635
LOC440345	-0.655
RPPH1	-0.661
LOC648509	-0.667
GNL3L	-0.671
LOC642033	-0.673
HS.163752	-0.673
C14ORF78	-0.683
LOC100134584	-0.694
LOC100133402	-0.695
ALKBH8	-0.695
NDUFS1	-0.696
HS.555252	-0.702
LOC100132774	-0.706
ZFHX3	-0.715
AAK1	-0.717
LOC646697	-0.724
GNB4	-0.725
SF1	-0.725
DLC1	-0.726
TOP3A	-0.743
HS.580797	-0.749
**Gene Symbol**	**Log Fold Change**
EYA4	-0.768
C9ORF38	-0.773
FAM129A	-0.777
HS.184721	-0.777
LATS2	-0.799
LOC652330	-0.801
LOC727987	-0.809
SLIT2	-0.809
C3ORF34	-0.818
UHMK1	-0.830
PHAX	-0.831
HS.572444	-0.832
RNU1F1	-0.845
TRQ1	-0.847
ZMAT3	-0.853
HIPK2	-0.866
TNFAIP8L1	-0.899
RNU1-3	-0.905
RNU1A3	-0.927
RNU1G2	-0.939
MIR886	-1.034
RNU4ATAC	-1.053
LOC100130516	-1.077
VTRNA1-1	-1.108
TRK1	-1.116
RNU1-5	-1.183
TDP1	-1.316
LOC100130835	-1.355
MIR1974	-1.570
RNY1	-1.702
SNORD13	-1.778
BCYRN1	-1.779
HS.579631	-1.965
HS.543887	-2.090
